# Changes in the proportion of facility-based deliveries and related maternal health services among the poor in rural Jhang, Pakistan: results from a demand-side financing intervention

**DOI:** 10.1186/1475-9276-10-57

**Published:** 2011-11-30

**Authors:** Sohail Agha

**Affiliations:** 1 Population Services International, 1120 19th Street, NW, Suite 600 Washington DC 20036, USA

## Abstract

**Background:**

Demand-side financing projects are now being implemented in many developing countries, yet evidence showing that they reach the poor is scanty.

**Methods:**

A maternal health voucher scheme provided voucher-paid services in Jhang, a predominantly rural district of Pakistan, during 2010. A pre-test/post-test quasi-experimental design was used to assess the changes in the proportion of facility-based deliveries and related maternal health services among the poor. Household interviews were conducted with randomly selected women in the intervention and control union councils, before and after the intervention.

A strong outreach model was used. Voucher promoters were given basic training in identification of poor women using the Poverty Scorecard for Pakistan, in the types of problems women could face during delivery, and in the promotion of antenatal care (ANC), institutional delivery and postnatal care (PNC). Voucher booklets valued at Rs. 4,000 ($48), including three ANC visits, a PNC visit, an institutional delivery, and a postnatal family planning visit, were sold for Rs. 100 ($1.2) to low-income women targeted by project outreach workers. Women suffering from complications were referred to emergency obstetric care services.

Analysis was conducted at the bivariate and the multivariate levels. At the multivariate level, logistic regression analysis was conducted to determine whether the increase in institutional delivery was greater among poor women (defined for this study as women in the fourth or fifth quintiles) relative to non-poor women (defined for this study as women in the first quintile) in the intervention union councils compared to the control union councils.

**Results:**

Bivariate analysis showed significant increases in the institutional delivery rate among women in the fourth or fifth wealth quintiles in the intervention union councils but no significant changes in this indicator among women in the same wealth quintiles in the control union councils. Multivariate analysis showed that the increase in institutional delivery among poor women relative to non-poor women was significantly greater in the intervention compared to the control union councils.

**Conclusions:**

Demand-side financing projects using vouchers can be an effective way of reducing inequities in institutional delivery.

## Background

Progress in the decline of maternal mortality has been disappointingly slow in Pakistan [1,2]. Even well-funded maternal health projects have failed to increase skilled birth attendance among the poorest women [3]. The government has not moved beyond the traditional supply-side approach to the provision of maternal health services through public sector outlets. The inadequate status of existing public sector facilities in Pakistan, with poor management and the poor quality and availability of products and services has contributed to low service utilization rates [4,5]. The primary barriers to delivering in a health facility operate at the financial and family levels: poor women have a clear preference for maternal health services from the private sector, which are perceived to be of higher quality, but cannot afford to pay for delivery care; husbands or mother-in-laws often do not support delivery in a health facility [2]. Although at least two-thirds of Pakistani women who seek delivery care obtain it from the private sector [5,6], there has been little public sector investment in improving the quality of private sector maternal health services or in making them financially accessible to the poor.

In spite of the need to increase skilled birth attendance among poor women, a population segment of particular importance because of the clustering of maternal deaths among the poor, neither national level projects nor community-based interventions have shown significant increases in institutional delivery among this segment [1,3,7]. The experience of interventions that have tried to generate the demand for institutional delivery among the poor indicates that the problem cannot be solved simply by increasing the demand for service utilization [1,3,5]. Public-private partnerships and financial incentive schemes to enable pregnant women to utilize maternity care services are some of approaches that have been suggested as a potential solution [5,7]. Focused demand generation among the poorest women combined with lowering financial barriers to accessing institutional delivery care meeting quality standards are likely to be important in lowering maternal mortality in Pakistan. A systematic review strongly indicates that voucher programs have been successful in increasing the utilization of health services [8]. At the same time, the review found only modest evidence of voucher programs being able to target specific populations [8].

Recently published studies from Pakistan and Bangladesh provide stronger evidence of voucher programs being able to target the poor. One study assessed the impact of demand-side financing strategy on increasing the use of maternal health services among low-income women in the urban center of one of the least developed districts in the Punjab: the study found that institutional delivery increased by 22 percentage points among voucher recipients; there was a substantial decline in the differential in institutional delivery between the fifth and the first quintiles [9]. A study in Bangladesh which analyzed the effects of a "universal" voucher scheme targeting pregnant women of parity 1 and 2 from all socio-economic groups found that the scheme's implementation was associated with reduced inequity in institutional delivery. Moreover, poor women were substantially more likely to take advantage of the voucher scheme than non-poor women [10].

This study assesses the impact of a demand-side financing intervention that sought to increase institutional deliveries among poor women (defined for this study as women in the two poorest quintiles) in a predominantly rural district of the Pakistani Punjab. The intervention addressed both financial and non-financial barriers to delivery care. The study determines whether the decline in wealth-based differentials in utilization of maternal health services was greater in the intervention than in the control area during the period in which the intervention was implemented.

## Methods

### The setting: Jhang

Located in central Punjab, Jhang is an agricultural district of the Punjab, with 77% of its population living in rural areas. In 2010, it had an estimated population of 2.4 million people living in 84 union councils [11]. Based on the availability of education, housing and employment, Jhang is one of the most deprived districts of the Punjab [12], with 59% of the population being below the poverty line [13].

A household survey conducted in 20 union councils of Jhang in 2009 showed that 41% of women delivered in a health facility [2]. The survey showed that differentials in institutional delivery were substantial: 61% of women in the first (wealthiest) quintile delivered in a health facility compared to 29% of women in the fifth (poorest) quintile. Further analysis showed that these wealth-based differentials remained significant even after controlling for mother's education and a range of other variables [2]. These findings were consistent Pakistani women's reports of the lack of affordability being one of the two main reasons for not delivering in a health facility [6]

In August 2009, a demand-side financing project was initiated. Outreach workers were hired and trained in promoting the use of maternal health services in 11 union councils of Jhang representing an estimated population of 330,000. Highly subsidized services, which could be accessed via vouchers, were delivered between January 2010 and December 2010. The project was funded by Population Services International through the Innovations Fund. The objective of the project was to determine whether a demand-side financing intervention could increase the utilization of ANC, PNC, institutional delivery, and family planning among poor women in Jhang.

### The intervention

The Jhang voucher scheme aimed to provide a package of maternity care and family planning services to 4,000 women in the two poorest quintiles in Jhang district. Based on the crude birth rate of 32.3 per 1,000 population for rural areas of Pakistan [6] and an estimated project area population of 330,000, we expected 10,659 births to occur in the intervention union councils during 2010. The package of services available to study participants included three ANC visits, a normal delivery visit combined with a PNC visit, a referral for a caesarian-section, a PNC visit following the caesarian-section and a postnatal family planning visit. Each service was provided through a coupon in the voucher booklet. Providers who received caesarian-section referrals were reimbursed by the project. The family planning visit could be used by the client to receive counseling or to receive a method. The booklet also contained two coupons for maternal tetanus shots which were to be provided when the mother came for an ANC visit and two coupons to enable clients to get their complete blood count and an ultrasound examination.

Many rural areas of Pakistan such as Jhang suffer from limited availability of skilled maternal health providers. Public sector facilities such as Basic Health Units, Rural Health Centers, Tehsil and District headquarter hospitals provide maternal and child health services but are often underutilized for a variety of reasons including inadequate management, non-availability of female staff, shortage of medicine, low motivation levels of service providers and restricted hours of operation (office hours for public sector providers are from 8 am to 2 pm) [5]. Some providers establish their private practice after completing several years of service in the public sector. Other public sector providers maintain a private practice during their off-duty hours. Although public sector facilities are supposed to provide services for free, the vast majority of public facilities charge for services. Private facilities operate on an explicit fee for service basis. Pakistani women who can afford to pay for services exhibit a clear preference for the private sector: more than two thirds of women who have an institutional delivery, deliver in a private health facility [4,6].

Providers selected for the voucher scheme were part of a network managed by Greenstar Social Marketing, a Pakistani NGO with a network of 7,500 participating providers trained in the provision of ANC, PNC, obstetric care, neonatal care, child care, and family planning services [14]. Based on an assessment of their structural capacity to provide quality maternal health services (facility cleanliness, infection prevention procedures, equipped labor room) providers were pre-approved for provision of services under this scheme. Twenty-three of the providers participating in the project were Lady Health Visitors (paramedics with 18 months of training in primary health care service provision, including maternity care) and five were physicians trained to conduct caesarian sections. Seven of the 28 providers also practiced in the public sector. A quality improvement program was initiated with a quality improvement officer (an experienced Lady Health Visitor) using structured instruments to observe and assess the quality of ANC, PNC and family planning service provision and training providers in the use of the partograph. After the initial assessment of the quality of service provision, the quality improvement officer made monthly supportive supervision visits during which she observed care provision and discussed how providers could improve the quality of care provided. To ensure sufficient reach of voucher-paid services within the project area, the primary basis for the selection of union councils for the project was the presence of Greenstar-trained providers.

No mass media was used to promote the vouchers. Promotion consisted of home visits by outreach workers during which outreach workers talked to pregnant women about the importance of institutional delivery and follow-up of women during and after pregnancy. The "product" was the package of maternal health services provided to poor women by eliminating the "monetary price" of the services offered, and by providing social support to the woman and her family to allow the delivery to occur in a health facility. Voucher booklets were distributed by outreach workers to women who met the poverty selection criteria established by the project. Thirteen project outreach workers, women with matriculate or higher level education who had previously worked as school-teachers or had worked for other NGOs, conducted the identification of voucher recipients using a tool developed by the World Bank for Pakistan. The Poverty Scorecard for Pakistan asks 13 questions on ownership of assets (e.g. agricultural land, livestock), possessions (e.g. motorcycle, refrigerator) amenities (e.g. toilet facilities), education (education of household head and school attendance of children) and the dependency ratio [15]. The maximum score that can be obtained by a household on the scorecard is 100 points; households which score 24 points or below are considered poor. A slightly less stringent standard was used for the selection of voucher recipients for this project in order to reduce the cost of identification (both in terms of the time and the resources that would be needed to contact a larger number of households if a stricter criterion were used): pregnant women scoring 33 points or below were considered eligible for the voucher booklet. Eligible women could purchase voucher booklets for a nominal price of Rs. 100 ($1.20).

Each of 13 outreach workers was given a target of identifying about 300 pregnant women who would be eligible to purchase the highly subsidized services offered by the project. To identify poor households, outreach workers conducted a rapid identification survey of households using the Poverty Scorecard in project union councils between mid-August and mid-November 2009. Voucher distribution began in mid-November. The identification survey was continued until 300 pregnant women were identified by each outreach worker. After being identified as a potential voucher recipient, a pregnant woman was informed about the voucher scheme and asked about her willingness to purchase a booklet for Rs. 100 (US$ 1.20) which would provide her a package of maternity services valued at Rs. 4,000 ($48) at no additional cost.

The sale of booklets was planned after the identification of eligible women. Hence, information on women interested in purchasing vouchers was entered into a Pregnant Woman Registration form. Sale of voucher booklets to eligible women began in November 2009. Multiple visits needed to be made to households with largely uneducated rural women who did not have previous exposure to such a scheme to explain how the voucher scheme functioned and to persuade them to deliver in a health facility.

The voucher booklet contained coupons for services that clients were entitled to receive upon purchase of the booklet. Each page of the voucher booklet had two identical, serialized coupons, one of which would be torn off by the provider and submitted to Greenstar after the service had been provided. The second coupon would remain attached to the booklet as a record of service utilization. Greenstar would reimburse providers at an agreed-upon rate for individual services within a month of coupon submission. Providers were paid a service charge of Rs. 100 for each of three ANC visits and Rs. 2,200 ($26) for a combined normal delivery and PNC visit. The provider payment for the PNC visit was combined with the payment for a normal delivery in order to encourage providers to keep a new mother at the facility for 24 hours after delivery-a high risk period for both the mother and the newborn. The provider payment for the caesarian delivery was Rs. 10,800 ($135). A separate PNC visit was advised/paid-for in the case of a caesarian-section. The provider service charge for the family planning visit was Rs. 100 ($1.2). Women who received services were reimbursed for their transportation cost by the provider at the following rates: Rs. 100 (US$1.2) for each ANC visit, Rs. 500 ($6.0) for normal delivery, Rs. 1,200 ($14.3) for a caesarian-section, and Rs. 150 ($1.8) for a family planning visit. A client could be counseled about birth spacing and receive a reversible contraceptive method such as an oral contraceptive, an injectable, a condom or an intrauterine device (IUD) through the coupon. Provider reimbursements for other services were as following: Rs. 50 for each of two tetanus vaccinations, Rs. 200 for a complete blood count, Rs. 150 for an ultrasound checkup. Greenstar reimbursed providers for transportation payments made by providers to women who received services. For example, the total reimbursement by Greenstar to a provider for an ANC visit was Rs. 200 ($2.4), which included Rs. 100 for the provider's service charge and Rs. 100 for the transportation reimbursement made by the provider to the woman who received the ANC service. If a tetanus shot was provided at the time of the ANC visit, the total reimbursement was Rs. 250 ($2.9).

Supervisors determined the validity of provider claims by randomly selecting and visiting the homes of women who had received voucher-paid services to determine whether they had indeed received services and whether they had been reimbursed for transportation costs by the provider. Three Field Supervisors and the Project Manager used Lot Quality Assurance Sampling to randomly select women who had received services through the project for follow-up. This enabled the project to check the veracity of a sample of claims at the time that claims were submitted. The Greenstar finance department transferred funds directly to provider bank accounts after the approval of individual claims.

Two follow-up contacts were also made by outreach workers to determine whether booklet purchasers were using voucher-paid services. The first of these was in July-August 2010. All 4,000 women who had been sold voucher booklets were re-contacted and asked about which services they had used. Their satisfaction with the services used was determined. Women who could still utilize unused coupons were encouraged to use the remaining services. The second follow-up contact was made in October-November 2010. Project MIS records showed high redemption rates for coupons: 97% of ANC 1 coupons, 91% of ANC 2 coupons, 85% of ANC 3 coupons, 94% of Tetanus 1 coupons, 80% of Tetanus 2 coupons, 86% of laboratory test coupons, 88% of ultrasound coupons and 96% of delivery coupons. By comparison, the use of the family planning coupon was much lower: 62% of the 4,000 women who were sold voucher booklets made the family planning visit after delivery.

In September 2010, a survey was commissioned to an independent market research agency to determine the validity of coupon utilization information in the MIS. Using the addresses of voucher recipients in the project MIS, interviewers from the research agency visited the homes of 550 women out of 3,000 women who had delivered according to project records by September 2010. The research agency was able to contact 533 women out of the 550 women-in other words, 97% of the sample from the MIS was verified through the survey. Of the women contacted by the research agency, 97% confirmed that they had delivered in a health facility during the last year, 95% reported that the provider had paid them for their transportation to the facility and 84% reported that they were very satisfied with the services received.

### Study design

The study was designed to determine whether the increase in service utilization was greater among poor women (i.e. those in the fourth or fifth quintiles) than among non-poor women (i.e. those in the first quintile) in intervention compared to control union councils. A union council is the smallest administrative unit in Pakistan with a population usually varying between 25,000 and 30,000. The study does not assume that all deliveries among poor women were through the use of vouchers. Indeed, the baseline household survey shows that about one-third of poor women were delivering in a health facility prior to the start of the voucher scheme. The study does assume that significant changes in institutional delivery among poor women in the intervention union councils, when not matched by significant changes among poor women in the control union councils, reflect the effects of the voucher scheme.

A pre-test/post-test quasi-experimental design was used to assess the impact of the project on service utilization. The assessment compares intervention and control union councils in terms of changes in the utilization of maternal health services during the 12-month period in which voucher-paid services were provided to the 12-month preceding the voucher scheme. Household survey data was collected in intervention and control union councils. Informed consent was obtained from female respondents prior to interviewing them, consistent with IRB procedures (IRB reference number 09-144129) approved by Tulane University Medical Center IRB.

### Data collection

The baseline household survey was conducted from November 16, 2009 to December 10, 2009. A five-days training was given to interviewers prior to data collection. This included classroom training, mock interviews and supervised practice interviews in the field. Data was collected from 10 intervention union councils and 10 adjacent union councils serving as controls.

Within each union council, multiple random starting points were chosen, and households were listed prior to the selection of eligible respondents. At the household level, a listing was done of married women 15-49 years who delivered in the last 12 months. A Kish-grid was used to randomly select one mother from each household. Using this approach, one hundred mothers who had delivered in the last 12-months were randomly selected from each union council. In total, 2,018 mothers were interviewed for the baseline study.

The same approach was used to collect the follow-up household survey data. The follow-up survey was conducted from December 8, 2010 to January 8, 2011 in the same 20 intervention and control union councils. A total of 2,033 mothers were interviewed for the follow-up study.

There was little variation in the population sizes of the 20 intervention and control union councils. Accordingly, no weights were attached to the data. Both surveys were conducted by AcNielsen Pakistan (Pvt.) Ltd. who have been conducting household surveys in Pakistan since 1991.

### Measures

#### Dependent variables

Four dependent variables were used for this analysis-ANC use, institutional delivery, PNC use, family planning use. For ANC use, women were coded '1' if they made at least three ANC visits, and '0' if they made fewer visits. For institutional delivery, women were coded as '1' if they delivered at a health facility and coded '0' if they delivered at home. For PNC use, women were coded '1' if they made a PNC visit, and '0' if they did not. For family planning use, women were coded '1'if they were using family planning at the time of the survey and '0' otherwise.

#### Independent variables

All independent variables included in the analysis are supported by prior literature on the determinants of ANC use, institutional delivery, PNC use and family planning use and were significant predictors of these outcomes in Jhang [2] and elsewhere in the Punjab [9]. Variables included in the analysis of ANC, institutional delivery, PNC and family planning are mother's age (categorized as 24 years or younger, 25-29 years, and 30 years or older), parity (number of living children), mother's education (none, primary, middle, secondary, matriculate or higher), wealth quintiles, travel time to the nearest health facility (within 15 minutes), and frequency of television viewership (daily or less often).

Wealth quintiles were created in a manner similar to their creation for the Demographic and Health Surveys [16]. Binary variables were created for the following household possessions and amenities: television, washing machine, refrigerator, bicycle, motorcycle, cell phone, source of water supply (a motorized hand-pump inside house versus other sources), flush connection to sewerage or septic tank, presence of a separate cooking area/kitchen in the house. Factor analysis was used to create a wealth factor score, which was divided into quintiles.

### Statistical analysis

#### Hypothesis testing

Logistic regression was used for the analysis. Models were tested to determine whether differentials in utilization of each of the services (ANC, delivery at a health facility, PNC, family planning) between the bottom two quintiles and the first quintile diminished more in the intervention area than in the control area between baseline and follow-up surveys. Independent variables were introduced in stages. The first model, Model 1, included dummy variables for wealth quintiles, a variable indicating whether the respondent belonged to the intervention or control group, and a variable indicating whether the respondent was interviewed pre-intervention or post-intervention. This model shows differentials in service utilization between the fifth and the first quintiles, and between the fourth and the first quintiles prior to adjusting for other variables. Model 2 adjusts for maternal factors (age, parity, maternal education) and other factors (travel time to the nearest health facility and frequency of television viewership). Model 3 includes an interaction between intervention group, time, and fifth quintile-which tests the null hypothesis that the change in service utilization between women in the fifth and the first quintiles is not significantly different between intervention and control union councils. The finding of a statistically significant difference will result in the rejection of the null hypothesis. Similarly, Model 4 includes an interaction which tests the null hypothesis that the change in service utilization between the fourth and the first quintiles is not significantly different between intervention and control union councils. The multi-stage design of the survey is taken into account in the statistical analysis by using the cluster option in STATA 10 [17].

## Results

Table 1 shows the characteristics of respondents in the intervention and control union councils at baseline. About two-fifths of women in the sample were under 25 years of age. Slightly more than one-fifth had one child and about one-fifth had five or more children. There were no significant differences between women in the intervention and control union councils in terms of age or parity. However, respondents in the intervention union councils were significantly different from respondents in the control union councils in terms of their education, travel time to the nearest facility, and television viewership: a smaller proportion of women in the intervention area had no education (58% vs. 69%, p < 0.001); women in the intervention area were more likely to live near a health facility (53% vs. 48%, p < 0.05); daily television viewership was higher in the intervention union councils (21% vs. 11%, p < 0.001).

**Table 1:** Characteristics of Respondents in Intervention and Control Union Councils at Baseline

	Intervention (n = 1,008)	Control (n = 1,010)	p-value
Mother's Age			
24 or younger	41.3	39.5	NS
25-29	34.7	33.2	
30 or older	24.0	27.3	
			
Parity/Living children			
1	24.4	22.4	NS
2	23.0	24.0	
3	19.4	18.2	
4	14.5	13.1	
5 or more	18.7	22.4	
			
Mother's education			
None	58.2	69.5	< 0.001
Primary	7.6	6.5	
Middle	12.4	11.1	
Secondary	8.9	7.4	
Matriculate or higher	12.8	5.4	
			
Travel time to nearest health facility			
More than 15 minutes	46.9	52.3	< 0.05
15 minutes or less	53.1	47.7	
			
Television viewership			
Never or sometimes	78.5	88.6	< 0.001
Daily	21.5	11.4	
			
Wealth quintiles			
Fifth/poorest	19.9	23.1	NS
Fourth	19.2	19.7	
Third	19.9	18.1	
Second	19.8	19.7	
First/non-poor	21.0	19.4	
			
	100.0	100.0	

*p < 0.05, **p < 0.01, ***p < 0.001, NS = not significant.

Table 2 compares changes in service utilization by wealth quintiles in intervention and control union councils. ANC use (3+ visits) increased by 15 percentage points among women in the fifth (poorest) quintile (from 23% to 39%, p < 0.01) in the intervention area but did not change in the control area. ANC use increased by 18 percentage points (from 31% to 49%) among women in the fourth quintile in the intervention union councils but did not change in the control union councils.

**Table 2:** Changes in ANC, institutional delivery, PNC, and family planning use by household wealth

	Intervention			Control		
	**Baseline** **%** **(n = 1,008)** **(1)**	**Follow-up** **%** **(n = 1,014)** **(2)**	**p-value for chi-square test** **(3)**	**Baseline** **%** **(n = 1,010)** **(4)**	**Follow-up** **%** **(n = 1,019)** **(5)**	**p-value for chi-square test** **(6)**

**ANC (3+ visits)**						
Wealth quintiles						
Fifth/poorest	23.4	38.6	< 0.01	20.6	19.2	NS
Fourth	30.9	49.5	< 0.001	20.6	27.0	NS
Third	37.3	49.8	< 0.05	27.9	30.2	NS
Second	46.5	58.1	< 0.05	32.7	44.5	< 0.05
First/non-poor	59.0	63.3	NS	46.4	58.6	< 0.05
						
**Institutional Delivery**						
Wealth quintiles						
Fifth/poorest	30.8	46.8	< 0.01	25.3	26.9	NS
Fourth	36.6	57.7	< 0.001	30.7	37.8	NS
Third	45.8	53.5	NS	29.5	44.8	< 0.01
Second	48.0	59.1	< 0.05	40.2	43.5	NS
First/non-poor	63.7	62.8	NS	55.1	56.5	NS
						
**PNC**						
Wealth quintiles						
Fifth/poorest	7.0	13.2	< 0.05	9.0	8.2	NS
Fourth	12.4	23.2	< 0.01	11.1	9.7	NS
Third	15.9	21.7	NS	12.6	14.1	NS
Second	17.5	24.7	NS	18.1	17.0	NS
First/non-poor	34.4	32.2	NS	28.6	25.3	NS
						
**Family Planning**						
Wealth quintiles						
Fifth/poorest	7.0	10.0	NS	6.9	9.0	NS
Fourth	7.7	11.9	NS	11.1	10.2	NS
Third	11.9	11.3	NS	8.2	12.5	NS
Second	11.5	21.1	< 0.01	11.6	11.5	NS
First/non-poor	18.4	20.1	NS	18.9	16.1	NS

NS = not significant.

A broadly similar pattern was observed for institutional delivery. The level of institutional delivery among women in the fifth and fourth quintiles increased by 16 percentage points (from 31% to 47%, p < 0.01) and 21 percentage points (from 37% to 58%, p < 0.001), respectively, in the intervention union councils but did not change in the control union councils.

PNC use increased among poor women in the intervention area. Women in the fifth quintile experienced a 6 percentage point increase in PNC use (from 7% to 13%, p < 0.05) and women in the fourth quintile experienced a 13 percentage point increase in PNC use (from 12% to 23%, p < 0.01). No change in PNC use was observed among women in the same wealth quintiles in the control union councils.

There was no evidence of an increase in family planning use among women in the fourth or fifth quintiles in the intervention union councils.

Table 3 shows odds ratios associated with institutional delivery in Jhang district. The findings are from logistic regression analyses. Model 1 tests the null hypothesis that there is no difference in institutional delivery between the poor (women in the fifth or fourth quintiles) and the non-poor. Prior to controlling for maternal education and other factors (Model 1), women in the fifth quintile had an odds ratio of delivering in a health facility which was one-third that of women in the first quintile (odds ratio = 0.32, p < 0.001). Women in the fourth quintile had half the odds of delivering in a health facility of women in the first quintile (odds ratio = 0.46, p < 0.001). These findings result in the rejection of the null hypothesis: there are significant differentials in institutional delivery between the poor and non-poor.

**Table 3:** Odds of Institutional Delivery in Jhang District, Pakistan (n = 4,051)

	Model 1	Model 2	Model 3	Model 4
Wealth quintiles				
Fifth/poorest	0.32***	0.56***	0.51***	0.57***
Fourth	0.46***	0.76*	0.76*	0.67***
Third	0.51***	0.79*	0.79*	0.79*
Second	0.61***	0.83	0.83	0.84
First/non-poor	1.00	1.00	1.00	1.00
				
**Intervention Group and Time of Survey**				
Group				
Control	1.00	1.00	1.00	1.00
Intervention	1.63*	1.53	1.47	1.45
				
Time/month of Survey				
Pre-intervention (December 2009)		1.00	1.00	1.00
Post-intervention (December 2010)	1.42***	1.43***	1.38***	1.36***
				
**Maternal Factors**				
Mother's Age				
24 or younger		1.00	1.00	1.00
25-29		1.15	1.15	1.15
30 or older		1.44***	1.44***	1.44***
				
Parity/Living children				
1		1.00	1.00	1.00
2		0.63***	0.63***	0.62***
3		0.47***	0.47***	0.47***
4		0.37***	0.37***	0.37***
5 or more		0.32***	0.32***	0.32***
				
Mother's education				
None		1.00	1.00	1.00
Primary		1.33*	1.32*	1.33*
Middle		1.30*	1.30*	1.31*
Secondary		1.96***	1.95***	1.97***
Matriculate or higher		2.43***	2.44***	2.46***
				
**Factors unrelated to the voucher scheme**				
Travel time to nearest health facility				
More than 15 minutes		1.00	1.00	1.00
15 minutes or less		1.03	1.03	1.03
				
Television viewership				
Never or sometimes		1.00	1.00	1.00
Daily		1.23*	1.24*	1.23*
				
Interaction 1 (intervention*time*5th quintile)			1.41*	
Interaction 2 (intervention*time*4th quintile)				1.64**

*p < 0.05, **p < 0.01, ***p < 0.001.

Model 2 tests the null hypothesis that differentials in institutional delivery between the poor and non-poor disappear after controlling for education and access to services. After controlling for maternal factors such as age, parity, education and factors such as travel time to the nearest health facility and frequency of television viewership (Model 3), differentials in institutional delivery declined: the odds of a woman in the fifth quintile delivering in a health facility were about half those of women in the first quintile (odds ratio = 0.56, p < 0.001); the odds of a woman in the fourth quintile having an institutional deliver were about three-quarters those of women in the first quintile (odds ratio = 0.76, p < 0.05). Thus, while some of the effects of being in the fourth or fifth quintiles on institutional delivery could be explained by the lower education and higher parity of women in these quintiles, poverty had an independent effect on institutional delivery. These findings lead to the rejection of the second hypothesis: differentials in institutional delivery between the poor and non-poor are independent of the lower education of the poor or lower access to services among the poor.

Model 3 tests the hypothesis that the change in institutional delivery between women in the fifth quintile and women in the first quintile was not different between intervention and control union councils. The finding of a statistically significant, positive odds ratio would lead to the rejection of the null hypothesis. Indeed, the significant and positive interaction term in Model 3 (odds ratio = 1.41, p < 0.05) indicates that the differential in institutional delivery between women in the fifth and first quintiles declined significantly faster in the intervention than in the control union councils: these findings leads to the rejection of the null hypothesis.

Model 4 tests the hypothesis that the change in institutional delivery between women in the fourth quintile or women in the first quintile was not significantly greater in intervention than in control union councils. The significant positive interaction term in Model 4 (odds ratio = 1.64, p < 0.01) indicates that the differential in institutional delivery between women in the fourth and the first quintile declined significantly faster among women in the intervention union councils than among women in the control union councils. In other words, these findings lead to a rejection of the null hypothesis.

The first panel in Table 4 shows the odds ratios associated with three or more ANC visits. Model 1 shows the substantial differentials in utilization of ANC between women in the bottom two quintiles and women in the first quintile. Model 2 shows that the differentials in utilization of ANC declined after controlling for age, parity, maternal education, travel time to the nearest facility, and daily television viewership but remained significant: women in the fifth quintile had half the odds of women in the first quintile of making three ANC visits (odds ratio = 0.50, p < 0.001); women in the fourth quintile had two-thirds the odds of women in the first quintile of making three or more ANC visits (odds ratio = 0.63, p < 0.01). Models 3 and 4 in Table 4 test the hypothesis of no difference in the change in institutional delivery between intervention and control union councils. The lack of significance of the interaction term in Model 3 does not allow rejection of the null hypothesis: in other words, the differential in ANC use among women in the fifth quintile relative to women in the first quintile did not decline faster in the intervention than in the control union councils. However, the interaction term in Model 4 was statistically significant (odds ratio = 1.64, p < 0.01), showing that the differential in ANC use between women in the fourth quintile and women in the first quintile declined faster in the intervention than in the control union councils.

**Table 4:** Odds of Antenatal Care and Postnatal Care Use in Jhang District, Pakistan (n = 4,051)

ANTENATAL CARE				
	Model 1	Model 2^†^	Model 3^†^	Model 4^†^
Wealth quintiles				
Fifth/poorest	0.25***	0.50***	0.47***	0.50***
Fourth	0.35***	0.63**	0.64**	0.55**
Third	0.42***	0.68**	0.68**	0.69**
Second	0.62***	0.88	0.89	0.89
First/non-poor	1.00	1.00	1.00	1.00
				
**Intervention Group and Time of Survey**				
Group				
Control	1.00	1.00	1.00	1.00
Intervention	1.79***	1.63**	1.59**	1.55**
				
Time/month of Survey				
Pre-intervention (December 2009)	1.00	1.00	1.00	1.00
Post-intervention (December 2010)	1.52***	1.54***	1.50***	1.46***
				
Interaction 1 (intervention*time*5th quintile)			1.27	
Interaction 2 (intervention*time*4th quintile)				1.64**

POSTNATAL CARE				

	Model 1	Model 2^†^	Model 3^†^	Model 4^†^
**Wealth quintiles**				
Fifth/poorest	0.24***	0.42***	0.39***	0.42***
Fourth	0.38***	0.63**	0.63**	0.50***
Third	0.44***	0.67*	0.67*	0.67*
Second	0.55***	0.75*	0.75*	0.75*
First/non-poor	1.00	1.00	1.00	1.00
				
**Intervention Group and Time of Survey**				
Group				
Control	1.00	1.00	1.00	1.00
Intervention	1.41*	1.31	1.29	1.22
				
Time/month of Survey				
Pre-intervention (December 2009)	1.00	1.00	1.00	1.00
Post-intervention (December 2010)	1.17	1.19	1.17	1.10
				
Interaction 1 (intervention*time*5th quintile)			1.24	
Interaction 2 (intervention*time*4th quintile)				2.04**

*p < 0.05, **p < 0.01, ***p < 0.001.

^† ^Model controls for age, parity, mother's education, travel time to facility, and television viewership.

The second panel in Table 4 shows the odds ratios associated with making a PNC visit. As in the case of institutional delivery and ANC care, differentials in the utilization of PNC care between the bottom two quintiles and the first quintile were substantial before controlling for other factors (Model 1). After controlling for other factors, these differentials declined but remained statistically significant (Model 2). The lack of statistical significance of the interaction term in Model 3 shows that the change in PNC use between women in the fifth and first quintiles was no different between intervention and control union councils. However, the significance of the interaction term in Model 4 shows that the differential in PNC care between the fourth and first quintiles declined significantly faster in the intervention than in the control union councils (odds ratio = 2.04, p < 0.01)

## Discussion

This study shows that there were statistically significant increases in institutional delivery among poor women (defined as women in the fifth and fourth quintiles) in the intervention but not in the control union councils during the period that a demand-side financing intervention was implemented in Jhang. Moreover, the increase in the proportion of institutional deliveries among poor women (fifth or fourth quintiles) relative to non-poor women was significantly greater in the intervention union councils than in the control union councils. These findings enable us to conclude that the Jhang voucher intervention significantly reduced differentials in institutional delivery between the poor and non-poor in Jhang district.

Similar, but less powerful effects of the intervention were observed in the case of ANC and PNC use. In both instances the demand-side financing intervention significantly reduced differentials in use of services between women in the first and fourth quintiles. The effects of the intervention in reducing differentials in ANC and PNC care among women in the first and fifth quintiles were in the right direction but did not reach statistical significance. One possible reason for this might be that the voucher scheme did not use the strict poverty cutoff of 24 points (out of 100) recommended in the Pakistan Poverty Scorecard. The poverty cutoff used by the project was 33 points. Stricter adherence to the recommended poverty cutoff may have resulted in significant reductions in differentials between women in the first and fifth quintiles. Another possible reason might be that the very poorest women (i.e. those in the fifth quintile) may be less responsive to the offer of free ANC or free PNC visits than to the offer of free institutional delivery.

The intervention did not have any effect on the use of family planning among poor women. One explanation for this is that the voucher promoters were not trained to counsel poor women about the importance of family planning. Project MIS records show that utilization of the family planning coupon, which could be used after the delivery/PNC visit, was 62% among women who were sold voucher booklets. This is in spite of the fact that the transportation reimbursement for the family planning visit was larger than the transportation reimbursement for the ANC or PNC visits (Rs. 150 versus Rs. 100). By comparison, the utilization of the three ANC coupons was 97%, 90% and 85%. Utilization of delivery care was 97%. These findings are consistent with findings from multiple studies which show that the barriers to the adoption of family planning in Pakistan are primarily cultural [18-20]. Reduced financial barriers alone are unlikely to be sufficient to increase the use of family planning among poor women in Pakistan.

In a voucher scheme implemented by Greenstar in Dera Ghazi Khan district [9], in which PNC visits were not bundled with the normal delivery visit, the level of family planning visits after delivery was much higher (79% of women who purchased voucher booklets made a family planning visit in Dera Ghazi Khan versus 62% in Jhang). The higher level of family planning visits in Dera Ghazi Khan among voucher recipients may have been because a PNC visit makes a good bridge between a delivery visit and a family planning visit. In the Jhang intervention, the PNC visit was bundled with the delivery care visit with the expectation that providers would keep women at the facility for 24 hours. It was also thought that a woman would find it more convenient to stay at the facility following delivery than make a return visit for PNC. In spite of the additional Rs. 200 ($2.3) incentive given to providers to keep women at the facility for 24 hours following delivery and in spite of outreach workers' efforts to motivate providers to keep women at the health facilities for 24 hours after delivery, only 25% of women stayed at the facility for 24 hours after delivery. The general trend in the Punjab is for women to leave the facility within 2-4 hours of normal delivery. Larger incentives than the Rs. 200 to providers to have women stay for 24 hours at the facility may be needed to change this behavior. In addition to the incentive for providers, incentives may be needed for women delivering at the health facility to keep them at the facility for 24 hours or longer. The inconvenience of being away from their homes, of leaving other children and household chores behind may outweigh the perceived safety of being at the facility for at least 24 hours after delivery. Alternatives should also be considered to keeping women at a facility for at least 24 hours: newborn health visits to women's home after delivery may be easier to implement than keeping mothers at the facility for 24 hours or longer.

Although quality of service delivery was observed at baseline and during monthly visits by the quality assurance officer who used a structured instrument for the observation-an approach that has been shown to be effective in improving in quality of care in primary health care [21]-changes in observed quality were not tracked over time. Measurement of quality of care using standardized checklists should be a part of voucher interventions. Linking provider payments to quality of service provision may be another important tool to increase the effectiveness of voucher interventions.

As described earlier, the social marketing "product" was a combination of the package of free maternal health services, transport vouchers, and social support offered to the woman and her family by outreach workers who made home visits. In general, it took outreach workers several visits to a woman's home to explain how the voucher scheme worked and to sell a voucher. The presence of the outreach worker in the community for several months and her meeting with potential voucher recipients and their family members to explain the importance of delivering in a health facility was a powerful motivating force. While this study was not designed to determine whether the home visit in itself would have been sufficient to stimulate the observed behavior change or whether it was the combination of the home visit and the reduction of the financial barriers to institutional delivery that motivated changes in behavior, several factors suggest that the removal of the financial barrier was an essential factor: a multivariate analysis of the baseline survey in Jhang showed that, prior to the voucher scheme, visits to women's homes by the government's health workers (who are expected to increase the utilization of maternal health services) were not associated with an increase in institutional delivery [2]; the multivariate analysis found powerful differentials in the utilization of institutional delivery by wealth quintiles [2], indicating that financial barriers played a powerful role prior to the introduction of the voucher scheme.

## Conclusions

Demand-side financing interventions can be used to reduce inequities in maternal health service utilization in rural Pakistan by increasing the level of institutional delivery among the poor. An improved design of a voucher intervention which links provider payments to quality of service provision should be used to further increase the impact of the approach used in this experiment.

## Competing interests

The author is an employee of Population Services International and served as a Senior Technical Advisor to Greenstar Social Marketing between October 2008 and December 2011.

## Authors' contributions

SA was responsible for the design of the study, the data analysis and for the write-up of the report.
